# Seasonal changes in diel haul‐out patterns of a lacustrine ringed seal (*Pusa hispida saimensis*)

**DOI:** 10.1002/ece3.10264

**Published:** 2023-07-03

**Authors:** Marja Niemi, Milaja Nykänen, Vincent Biard, Mervi Kunnasranta

**Affiliations:** ^1^ Department of Environmental and Biological Sciences University of Eastern Finland Joensuu Finland; ^2^ Natural Resources Institute Finland Joensuu Finland

**Keywords:** camera trapping, circadian, conservation, diel, haulout, pinniped, Saimaa ringed seal, telemetry

## Abstract

Seasonal changes in diel haul‐out patterns of the lacustrine Saimaa ringed seal (*Pusa hispida saimensis*) were studied using a combination of satellite telemetry and camera traps during 2007–2015. We found the haul‐out activity patterns to vary seasonally. Our results show that during the ice‐covered winter period before the seals start their annual molt, the peak in haul‐out generally occurs at midnight. Similarly, during the postmolt season of summer and autumn when the lake is free of ice, the haul‐out is concentrated in the early hours of the morning. In contrast, over the spring molting period, Saimaa ringed seals tend to haul out around the clock. The spring molt is also the only period when a slight difference in haul‐out behavior between the sexes is observed, with females having a haul‐out peak at nighttime while the males have a less visible diel pattern. According to our results, the diel haul‐out patterns of Saimaa ringed seals are similar to the ones of marine ringed seals. Gathering information on haul‐out activity is important in order to safeguard the natural patterns of Saimaa ringed seals in areas that are prone to disturbance from human activities.

## Graphical abstract

Haul‐out activity patterns of Saimaa ringed seals (*Pusa hispida saimensis*) varied seasonally. Saimaa ringed seals are predominantly day‐active animals, resting nocturnally year‐round, but spending time hauled out during the day for molting purposes in the spring.

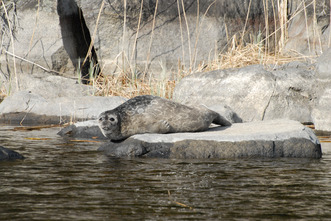

## 1 INTRODUCTION

Saimaa ringed seal (*Pusa hispida saimensis*) is an endangered subspecies endemic to Lake Saimaa in Eastern Finland. The population has been increasing in recent decades due to various conservation measures (Kunnasranta et al., 2021) and is nowadays around 440 individuals (Metsähallitus, 2022). The biggest threats are by‐catch, small population size, disturbance, and climate change (Kunnasranta et al., 2021). During the open‐water season, seals haul out and rest on terrestrial platforms, although they can also rest in water (Kunnasranta et al., 2002). However, haul out while sleeping may reduce metabolic costs, as seen in harbor seals (*Phoca vitulina*; Watts, 1996). During the ice‐covered season in winter, Saimaa ringed seals dig lairs in snow drifts that accumulate on the shorelines of islands and islets (Sipilä, 2003). These snowlairs are used for giving birth and nursing as well as for haul‐out. As with all other ringed seals, Saimaa ringed seals undergo annual molt during spring. However, unlike with ringed seals in the Arctic that typically molt on ice (Smith, 1973), the peak of the molt in Lake Saimaa takes place on terrestrial platforms after the ice breaks (Niemi et al., 2022) with individuals using the same molting sites year after year (Biard et al., 2022). Terrestrial haul‐out behavior is also seen in other subspecies, the Ladoga (*P. h. ladogensis*) and Baltic (*P. h. botnica*) ringed seals, which may complete their molt on terrestrial platforms (Härkönen et al., 1998; Popov, 1979). Also, Arctic ringed seals (*P. h. hispida*) have recently been observed to haul out on land in Svalbard due to lack of ice (Lydersen et al., 2017).

Haul‐out patterns of Saimaa ringed seals have been studied before with the emphasis on the period after the molt, and it has been found that during this period the haul‐out occurs mainly at night (Hyvärinen et al., 1995; Kunnasranta et al., 2002; Niemi et al., 2013). It is also generally known that Saimaa ringed seals haul out also during the day during molt. However, only recently there have been studies focusing specifically on the molting season (Biard et al., 2022; Niemi et al., 2022), and very little is known about haul‐out patterns before the molt during the ice‐covered season in winter. The extreme molting site fidelity (Biard et al., 2022) coupled with the fact that their molt coincides with the peak of seal‐watching activity (as they can easily be observed from boats) makes Saimaa ringed seals especially vulnerable to disturbance (Niemi et al., 2013, 2022). Therefore, adding to the information on diel haul‐out patterns during different stages of the seals' annual cycle is important so that potential effects of human disturbance can be evaluated. Thus, the objective of this study was to describe the seasonal changes in diel haul‐out patterns of Saimaa ringed seals.

## 2 MATERIALS AND METHODS

**FIGURE 1:**
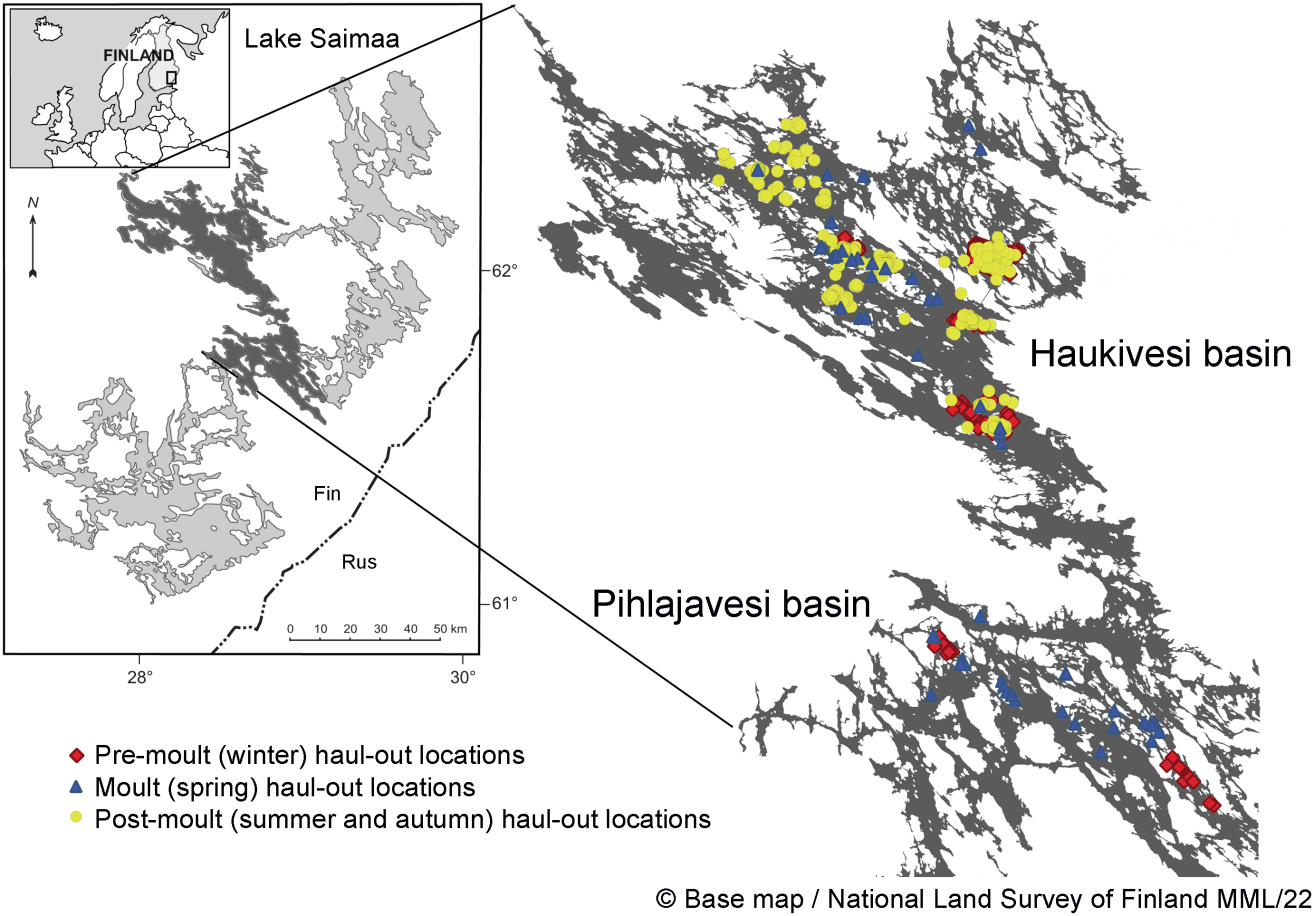
A map showing the haul‐out locations of studied Saimaa ringed seals during different seasons.

The study was carried out in Pihlajavesi and Haukivesi water basins of Lake Saimaa, Finland (Figure 1), where approximately half of the Saimaa ringed seal population lives (Metsähallitus, 2022). We combined camera trap and satellite telemetry data to estimate the seasonal variability in diel haul‐out patterns during (1) premolt in winter (from the start of ice cover around December until the satellite tags drop off), (2) peak of molt in spring (May–mid‐June), and (3) postmolt in summer and autumn (from mid‐June until the lakes freeze over) (Table 1 and Table S1).

**TABLE 1:** Description of the study design and the data of diel haul‐out patterns of the Saimaa ringed seal.

Season	Water basin	Seals (*N*)	Haul‐out events (*N*)	Study years	Study method
Premolt	HV & PV	7	778	2009–2015	Satellite tags (Niemi et al., 2019)
Molt	HV & PV	60	356	2012–2013	Camera traps (Niemi et al., 2022)
Postmolt	HV	8	1280	2007–2011	Satellite tags (Niemi et al., 2013)

Abbreviations: HV, Haukivesi; PV, Pihlajavesi.

Camera traps enabled monitoring during the molting season, while the tags, which are glued to pelage, drop off due to weakening of the bond to the hair or at molt at the latest. In this study, the last tag dropped off at the second week of April (Table S1), before the peak of the molting season (Niemi et al., 2022). The game cameras (*N* = 50 in 2012 and *N* = 63 in 2013, Scout Guard 550VB and 560K‐8M with 2–4 GB memory cards) were deployed on trees or wooden planks placed in the vicinity of observed haul‐outs. Due to the seals' molting site fidelity (Biard et al., 2022), most of the potential haul‐outs are well known beforehand although water level can cause slight variation on the precise location. We chose the final locations for the camera deployments after observations of seals made during boat surveys. Game cameras were set to operate continuously with motion‐sensitive triggers to take two to three photos over a 0.5–2 min time span, from May to mid‐June annually (Table 1). We used permanent pelage patterns to identify individual seals from the photographs (see Koivuniemi et al., 2016). Furthermore, the sex was determined visually from the photographs when possible. The telemetry data included haul‐out records from 12 satellite‐tagged adult individuals (see Niemi et al., 2012, 2019) from pre‐ and postmolt seasons in 2007–2015 (Table 1). For tagging, all the seals were captured in water near their haul‐out site in May–early June. GPS‐GSM transmitters (SMRU, University of St. Andrews, UK) were glued to the seal's dorsal postmolt pelage (see Niemi et al., 2012). The haul‐out was determined by the wet‐dry sensor of the tag; a haul‐out event started when the sensor was dry for 10 min and ended when the sensor became wet for 40 s. Thus, only haul‐out events lasting more than 10 min were included in the data.

For each hour of the day (local Finnish time), we quantified the haul‐out activity as the proportion of time in minutes the seal was hauled out. For example, if the seal was hauled out for 15 min in an hour, the haulout proportion/activity would be 0.25 for that hour. For camera trap data, we included each haul‐out longer than 10 min, to allow comparison with the satellite tag data.

Research was conducted under permits by the Finnish environmental authorities ELY center (ESAELY/433/07.01/2012, ESA‐2008‐L‐519‐254), Metsähallitus (MH 5813/2013 and MH 6377/2018/05.04.01) and the Project Authorisation Board (formerly Animal Experiment Board) in Finland (ESAVI/8269/04.10.07/2013 and ESAVI‐2010‐08380/Ym‐23).

To test whether the diel haul‐out patterns varied among different seasons and between sexes, we modeled the hourly haul‐out proportion using a Generalized Additive Mixed Model, GAMM (Lin & Zhang, 1999; Wood, 2017) with a Beta distribution. Because the data included a considerable number of hours when the proportion of haul‐out was zero (seals did not haul out at all during the hour) or one (seals hauled out for the entire hour), we applied the zero and one inflated version of this distribution, available in the “brms” package (Bürkner, 2017, 2018, 2021) that runs Bayesian models in Stan (Stan Development Team, 2022) via R (R Core Team, 2023). Season and sex were included in the model as fixed factors, and hour of the day, separately for each season and sex, was included as a circular cubic smooth (with *k* = 6 basis functions) using the “by” term from the “mgcv” package for GAMMs (Wood, 2011, 2017). Because it was possible to identify the seals individually, seal ID was included in the model as a random term in order to avoid pseudoreplication and to minimize possible overdispersion. We ran the models with a random intercept and a correlated random intercept and slope, and chose the best random effect structure by leave‐one‐out (LOO) cross‐validation (Pareto smoothed importance sampling approximation, PSIS, Vehtari et al., 2015, 2017). The PSIS‐LOO method (with 100 runs) was also used to decide whether to keep the fixed factors in the model. All four candidate models were run using four chains, 1000 iterations preceded by 1000 warm‐up steps, and with the default non‐informative priors. Convergence of chains was verified visually and based on the potential scale reduction factor on split chains (Rhat) values, and the independence of draws was determined based on the effective sample size (ESS) values. The fit of the models to the data was inspected by drawing 4000 replicate samples (*y*_rep_) from the posterior predictive distribution using “rstantools” (Gabry et al., 2023) and plotting the simulated distribution of a test statistic *T*(*y*_rep_), in this case, the mean proportion, over the simulated datasets in *y*_rep_ with the observed value *T*(*y*) (mean proportion) computed from the data *y* using “bayesplot” (Gabry & Mahr, 2022).

## 3 RESULTS

Premolt and postmolt telemetry data of four females and eight males consisted of 778 and 1280 haulout events, respectively. In addition, camera trap data consisted of 356 haul‐out events from 60 different individuals (31 females, 26 males, and three unknowns, Table 1 and Table S1).

**FIGURE 2:**
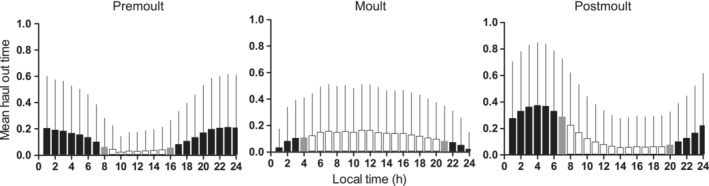
Seasonal haul‐out patterns of Saimaa ringed seals. Haul out activity (proportion of time in minutes) per each hour of local Finnish time (mean + SD) during premolt (*N* = 7 seals), molt (*N* = 60 seals), and postmolt season (*N* = 8 seals). White bars denote hours of daylight, dark bars the hours of darkness, and gray bars denote average sunrise and sunset times.

The seals hauled out mainly during nighttime in the pre‐ and postmolting seasons, whereas during the molt, haul‐out occurred throughout the day (Figure 2). All the candidate models converged well with Rhat values of 1.00–1.01 and all ESS values >850 (most values >1000). The expected log predictive densities from the PSIS‐LOO cross‐validation indicated that the best model included the random effect of seal ID as a correlated random intercept and slope and both factor variables season and sex. The plot of the simulated distribution *T*(*y*_rep_) with the observed value *T*(*y*) (mean proportion) indicated a well‐fitting model (Figure S1).

**FIGURE 3:**
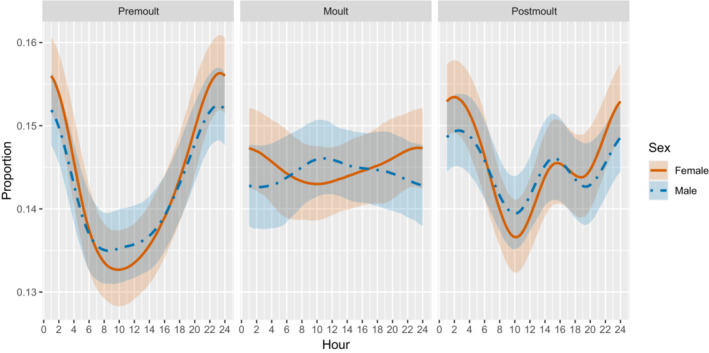
Conditional effects of the hour of the day on the proportion of haul‐out of Saimaa ringed seals during different seasons and with sex. The solid (females) and dashed (males) lines depict the model‐estimated mean and shaded areas 95% credible intervals.

The interaction between season and smooth of an hour of the day was significant with the premolt and postmolt seasons as the 95% credible intervals (CIs) of the posterior distributions did not contain zero (premolt: 0.16–1.24, postmolt: 0.15–1.23). However, during the molting season, the proportion of time the seals spent hauled out did not vary significantly by the hour of the day (95% CI: 0.00–0.44). The variation among individuals (random effect) was significant with the intercept standard deviation (SD) of 0.70 (95% CI: 0.52–0.94) and slope SD of 0.04 (95% CI: 0.03–0.06) across individuals. In general, the hourly proportion of haul‐out peaked at midnight (Finnish local time) during premolt, and at around 2 a.m. during postmolt (with a secondary peak at around 3 p.m.) whereas such a clear peak in the hourly proportion was less evident during the molting season (Figure 3). Interaction between sex and smooth of an hour was significant with females (95% CI: 0.01–0.54) but not with males (95% CI: 0.00–0.37). When comparing the haul‐out patterns between the sexes during the molting season, females concentrated their haul‐out more at nighttime whereas males showed a less visible diel pattern (Figure 3).

## 4 DISCUSSION

We found that the haul‐out patterns of Saimaa ringed seals varied with season. Haul‐outs occurred predominantly at night, but during the molt the seals switched to haul out around the clock. Our study, which is the first to combine data from all the stages of the annual cycle, was concordant with previous season‐specific studies on Saimaa ringed seals' diel behavior (Hyvärinen et al., 1995; Kunnasranta et al., 2002; Niemi et al., 2013, 2022). Such seasonal behavior is also observed in marine ringed seals (Hamilton et al., 2018; Härkönen et al., 2008; Kelly et al., 2010; Kelly & Quakenbush, 1990; Von Duyke et al., 2020), although some studies done in constant light regimes in the Arctic have also reported a lack of clear diel pattern, especially during periods of constant daylight and darkness (Born et al., 2002; Hamilton et al., 2018). The reason for extending the haul‐out also to daytime during molt is most likely physiological, as having warm and dry skin accelerates this process (Feltz & Fay, 1966). Molting is an energetically demanding period for the seals (Thometz et al., 2021), so concentrating haul‐out to the hours of most solar energy is advantageous.

Only during the molting period, there seems to be a slight difference in the haul‐out patterns between sexes. While males hauled out around the clock with a slight preference for daytime, females continued to have a peak in the haul‐out during nighttime. A correlation between air temperature and the number of molting females has been described before (Niemi et al., 2022), and this could at least partly explain the difference in the haul‐out patterns between the sexes during the molt. Diel rhythm can be defined as rhythmic alterations in physiological processes that enable the prediction of the changes in light and dark cycles, which allows mammals to perform their activities at the optimal time (Güldür & Otlu, 2017). The activity and resting patterns of mammals depend on a variety of factors related to weather, seasonal changes in light and temperature, prey behavior, predation risk, competition, and anthropogenic pressure (Cox et al., 2021). Also, the tidal cycle may have an impact on marine seals' haul‐out patterns (Thompson et al., 1997). The haul‐out of ringed seals is hypothesized to be tied to environmental factors (Carlens et al., 2006; Hamilton et al., 2018; Smith & Hammill, 1981) and the diel patterns also to behavior of their prey and predators (Hamilton et al., 2018; Härkönen et al., 2008; Kelly & Quakenbush, 1990; Von Duyke et al., 2020).

Our results showed a peak in the hourly proportion of haul‐out at midnight in winter. Also during summer and autumn, the peak was a few hours after midnight. Although the peak hours varied, there was no significant difference in the timing of haul‐out between these seasons. Saimaa ringed seals use terrestrial sites to haul out when there is no ice cover, and they have options to choose sheltered sites in the labyrinthic lake. Also, they are not susceptible to the tidal cycle unlike seals living in the marine environment. Furthermore, adults have no predators in the Saimaa region. Therefore, out of the possible biotic factors, the distribution and behavior of prey species is most likely to have an effect on the activity patterns, at least outside the molting season, although we cannot make a straightforward connection to this. Saimaa ringed seals feed mostly on small schooling fish (Auttila et al., 2014; Kunnasranta et al., 1999) and the prey diel activity patterns, such as changes in schooling behavior, vertical movements, and distribution (Jurvelius & Marjomäki, 2004), may have an effect on the seals' behavior patterns. It is also likely that human activity influences haul‐out patterns. Lake Saimaa is an important waterway for transport of goods and a hub for recreational boating and fishing. Moreover, there are almost 70,000 buildings including residential houses and summer cottages along the shoreline (Liukkonen et al., 2017). Saimaa ringed seals may choose to mainly haul out at night to avoid human disturbance (Kunnasranta et al., 2002; Niemi et al., 2013). However, the benefits of daylight (such as warmer temperatures) during the molting season likely overcome the cost of disturbance.

Although our satellite tracking and camera trap data sets were different, as telemetry provided many observations of a few individuals while camera traps rather targeted a larger number of individuals at certain sites, we are confident that the observed variation in diel patterns reflect the different stages in the animals' annual cycle rather than differences in data collection methods. Although it is possible that in some cases the haul‐out was interrupted by disturbance or camera failure, we do not think this has a large influence on the estimation of the overall diel pattern due to the vast amount of data collected (the game cameras recorded >7500 h). Different life stages of the seals may have a greater impact on the activity patterns. However, to our knowledge, our dataset does not include females that gave birth, and all individuals were adults or near adulthood based on their size. We cannot overrule the fact that some seals likely began their molt already during the ice‐covered season, and if the satellite tags dropped off due to molt, there may be a slight overlap with these two periods. Furthermore, it has been shown that the first weeks after the tags have been glued to the new hair also shows slightly different diel haul out patterns than later during the postmolt season (Niemi et al., 2012). However, the data clearly includes the peak times of each season and captures the variation between these seasons.

This study adds to the knowledge of Saimaa ringed seals' behavior patterns by demonstrating a clear diel haul‐out pattern that changes during the molt. The diel peaks of haul out are at early hours during postmolt and at midnight during premolt, whereas during the molt there is a less visible peak and the seals tend to haul out throughout the day. Changes in the haulout patterns of Saimaa ringed seals may signal a change in the level of human disturbance or other environmental changes that may have fitness consequences on this endangered subspecies.

## AUTHOR CONTRIBUTIONS

**Marja Niemi:** Conceptualization (equal); data curation (lead); formal analysis (equal); funding acquisition (supporting); investigation (equal); methodology (equal); project administration (equal); supervision (equal); validation (equal); visualization (equal); writing – original draft (lead); writing – review and editing (lead). **Milaja Nykänen:** Conceptualization (equal); data curation (supporting); formal analysis (lead); investigation (equal); methodology (equal); supervision (equal); validation (equal); visualization (equal); writing – original draft (equal); writing – review and editing (equal). **Vincent Biard:** Conceptualization (supporting); data curation (supporting); methodology (supporting); validation (equal); writing – original draft (equal); writing – review and editing (equal). **Mervi Kunnasranta:** Conceptualization (equal); data curation (equal); formal analysis (supporting); funding acquisition (lead); investigation (equal); methodology (equal); project administration (equal); supervision (lead); validation (equal); writing – original draft (equal); writing – review and editing (equal).

## CONFLICT OF INTEREST STATEMENT

The authors have no conflicts of interest to declare that are relevant to the content of this article.

## Supporting information


Appendix S1.

## Data Availability

The dataset generated and analyzed in the present study can be found as Supplementary Material.
