# Supplementary material for: Seasonal changes in diel haul‐out patterns of a lacustrine ringed seal (Pusa hispida saimensis)
Source: Ecol Evol. 2023 Jul 3;13(7):e10264. doi: 10.1002/ece3.10264 (PMC10316366; doi:10.1002/ece3.10264)
Supplement: Supplementary file 1 — Appendix S1. [file ECE3-13-e10264-s001.docx]

**Seasonal changes in diel haul-out patterns of a lacustrine ringed seal (*Pusa hispida saimensis*)**

Marja Niemi^1*^, Milaja Nykänen^1^, Vincent Biard^1^, Mervi Kunnasranta^1,2^

Supporting information

Table.S1. Description of the individual haul-out data collected by telemetry and camera traps for the different study seasons. F = female, M = male, UNK = unknown. HV = Haukivesi basin, PV = Pihlajavesi basin

|  |  | | Pre-moult | | | Moult | | | Post-moult | | |
| --- | --- | --- | --- | --- | --- | --- | --- | --- | --- | --- | --- |
| Seal ID | Sex | Water Basin | First haulout during season  (dd/mm/yyyy) | End (dd/mm/yyyy) | Total number of haulouts | First haulout during season  (dd/mm/yyyy) | End (dd/mm/yyyy) | Total number of haulouts | First haulout during season  (dd/mm/yyyy) | End (dd/mm/yyyy) | Total number of haulouts |
| TO07 (010) | M | HV | 19/12/2009 | 28/03/2010 | 37 | 22/05/2012 | 30/05/2012 | 7 | 17/06/2009 | 12/12/2009 | 151 |
| OL10 (031) | F | HV | 29/11/2010 | 02/04/2011 | 159 | 09/06/2012 | 13/06/2012 | 3 | 16/06/2010 | 15/11/2010 | 135 |
| TE07_11 | F | HV | 03/01/2012 | 02/02/2012 | 7 |  |  |  | 16/06/2011 | 30/12/2011 | 247 |
| AS12 | M | HV | 08/12/2012 | 10/01/2013 | 13 |  |  |  |  |  |  |
| VO12 | M | HV | 06/12/2012 | 02/04/2013 | 473 |  |  |  |  |  |  |
| MI13 (087) | M | PV | 25/01/2014 | 12/04/2014 | 53 | 27/05/2013 | 15/06/2013 | 10 |  |  |  |
| JE14 (104) | M | PV | 12/01/2015 | 12/02/2015 | 36 | 25/05/2013 | 28/05/2013 | 2 |  |  |  |
| 002 | F | HV |  |  |  | 16/05/2012 | 05/06/2012 | 14 |  |  |  |
| 005 | M | HV |  |  |  | 29/05/2012 | 04/06/2012 | 3 |  |  |  |
| 017 | M | HV |  |  |  | 18/05/2012 | 05/06/2012 | 11 |  |  |  |
| 018 | F | HV |  |  |  | 01/06/2012 | 01/06/2012 | 1 |  |  |  |
| 019 | F | HV |  |  |  | 09/06/2012 | 09/06/2012 | 1 |  |  |  |
| 021 | M | HV |  |  |  | 19/05/2012 | 13/06/2012 | 12 |  |  |  |
| 023 | M | HV |  |  |  | 16/05/2012 | 08/06/2012 | 20 |  |  |  |
| VI09 (026) | M | HV |  |  |  | 17/05/2012 | 30/05/2012 | 7 | 16/06/2009 | 09/12/2009 | 239 |
| 027 | F | HV |  |  |  | 25/05/2012 | 25/05/2012 | 1 |  |  |  |
| LI10 (028) | F | HV |  |  |  | 18/05/2012 | 01/06/2012 | 11 | 17/06/2010 | 14/07/2010 | 31 |
| 029 | F | HV |  |  |  | 16/05/2012 | 19/05/2012 | 4 |  |  |  |
| 034 | F | HV |  |  |  | 16/05/2012 | 11/062012 | 10 |  |  |  |
| 036 | F | HV |  |  |  | 17/05/2012 | 07/06/2012 | 4 |  |  |  |
| 038 | M | HV |  |  |  | 24/05/2012 | 25/05/2012 | 2 |  |  |  |
| 040 | M | HV |  |  |  | 20/05/2012 | 21/05/2012 | 2 |  |  |  |
| 042 | M | HV |  |  |  | 29/05/2012 | 29/05/2012 | 1 |  |  |  |
| 048 | M | HV |  |  |  | 18/05/2012 | 18/05/2012 | 1 |  |  |  |
| 049 | M | HV |  |  |  | 10/05/2012 | 09/06/2012 | 8 |  |  |  |
| 050 | F | HV |  |  |  | 21/05/2012 | 08/06/2012 | 7 |  |  |  |
| 051 | M | HV |  |  |  | 18/05/2012 | 13/06/2012 | 16 |  |  |  |
| 052 | F | HV |  |  |  | 19/05/2012 | 19/05/2012 | 1 |  |  |  |
| 053 | unk | HV |  |  |  | 26/05/2012 | 06/06/2012 | 28 |  |  |  |
| 061 | F | HV |  |  |  | 21/05/2012 | 07/06/2012 | 20 |  |  |  |
| 062 | F | HV |  |  |  | 22/05/2012 | 24/05/2012 | 3 |  |  |  |
| 064 | M | HV |  |  |  | 17/05/2012 | 21/05/2012 | 3 |  |  |  |
| 065 | F | HV |  |  |  | 22/05/2012 | 23/05/2012 | 3 |  |  |  |
| 066 | F | HV |  |  |  | 21/05/2012 | 31/05/2012 | 10 |  |  |  |
| 067 | F | HV |  |  |  | 06/05/2012 | 30/05/2012 | 5 |  |  |  |
| 082 | M | PV |  |  |  | 17/05/2013 | 15/06/2013 | 10 |  |  |  |
| 083 | M | PV |  |  |  | 24/05/2013 | 24/05/2013 | 1 |  |  |  |
| 084 | M | PV |  |  |  | 06/06/2013 | 06/06/2013 | 1 |  |  |  |
| 085 | F | PV |  |  |  | 26/05/2013 | 02/06/2013 | 8 |  |  |  |
| 086 | M | PV |  |  |  | 24/05/2013 | 28/05/2013 | 7 |  |  |  |
| 088 | F | PV |  |  |  | 19/05/2013 | 09/06/2013 | 3 |  |  |  |
| 090 | F | PV |  |  |  | 17/05/2013 | 18/05/2013 | 2 |  |  |  |
| 091 | F | PV |  |  |  | 23/05/2013 | 28/05/2013 | 5 |  |  |  |
| 092 | F | PV |  |  |  | 23/05/2013 | 24/05/2013 | 1 |  |  |  |
| 093 | M | PV |  |  |  | 04/05/2013 | 05/052013 | 1 |  |  |  |
| 094 | F | PV |  |  |  | 26/05/2013 | 04/06/2013 | 3 |  |  |  |
| 095 | F | PV |  |  |  | 10/05/2013 | 01/06/2013 | 4 |  |  |  |
| 096 | M | PV |  |  |  | 27/05/2013 | 27/05/2013 | 1 |  |  |  |
| 097 | F | PV |  |  |  | 17/05/2013 | 25/05/2013 | 4 |  |  |  |
| 098 | F | PV |  |  |  | 26/05/2013 | 04/06/2013 | 5 |  |  |  |
| 099 | M | PV |  |  |  | 14/05/2013 | 14/05/2013 | 1 |  |  |  |
| 100 | F | PV |  |  |  | 24/05/2013 | 04/06/2013 | 2 |  |  |  |
| 101 | M | PV |  |  |  | 28/05/2013 | 28/05/2013 | 1 |  |  |  |
| 102 | unk | PV |  |  |  | 26/05/2013 | 26/05/2013 | 1 |  |  |  |
| 103 | M | PV |  |  |  | 03/06/2013 | 16/06/2013 | 10 |  |  |  |
| 105 | M | PV |  |  |  | 19/05/2013 | 04/06/2013 | 19 |  |  |  |
| 106 | M | PV |  |  |  | 19/05/2013 | 28/05/2013 | 14 |  |  |  |
| 108 | unk | PV |  |  |  | 25/05/2013 | 13/06/2013 | 2 |  |  |  |
| 110 | M | PV |  |  |  | 15/05/2013 | 16/05/2013 | 2 |  |  |  |
| 111 | F | PV |  |  |  | 15/05/2013 | 16/05/2013 | 1 |  |  |  |
| 114 | F | PV |  |  |  | 08/06/2013 | 08/06/2013 | 1 |  |  |  |
| 115 | F | PV |  |  |  | 07/06/2013 | 12/06/2013 | 5 |  |  |  |
| 117 | M | PV |  |  |  | 04/06/2013 | 07/06/2013 | 2 |  |  |  |
| Unknown in place1 | unk | HV |  |  |  | 18/05/2012 | 01/06/2012 | 2 |  |  |  |
| Unknown in place2 | unk | HV |  |  |  | 19/05/2012 | 19/05/2012 | 1 |  |  |  |
| Unknown in place3 | unk | HV |  |  |  | 31/05/2012 | 01/06/2012 | 1 |  |  |  |
| Unknown in place4 | unk | HV |  |  |  | 31/05/2012 | 01/06/2012 | 1 |  |  |  |
| Unknown in place5 | unk | HV |  |  |  | 03/06/2012 | 05/06/2012 | 3 |  |  |  |
| HE07 (006) | F | HV |  |  |  |  |  |  | 16/06/2007 | 24/11/2007 | 179 |
| KJ07 (007) | M | HV |  |  |  |  |  |  | 16/06/2007 | 30/12/2007 | 179 |
| ER11 (012) | M | HV |  |  |  |  |  |  | 16/06/2011 | 20/09/2011 | 119 |
| Total |  |  |  |  | 778 |  |  | 356 |  |  | 1 280 |


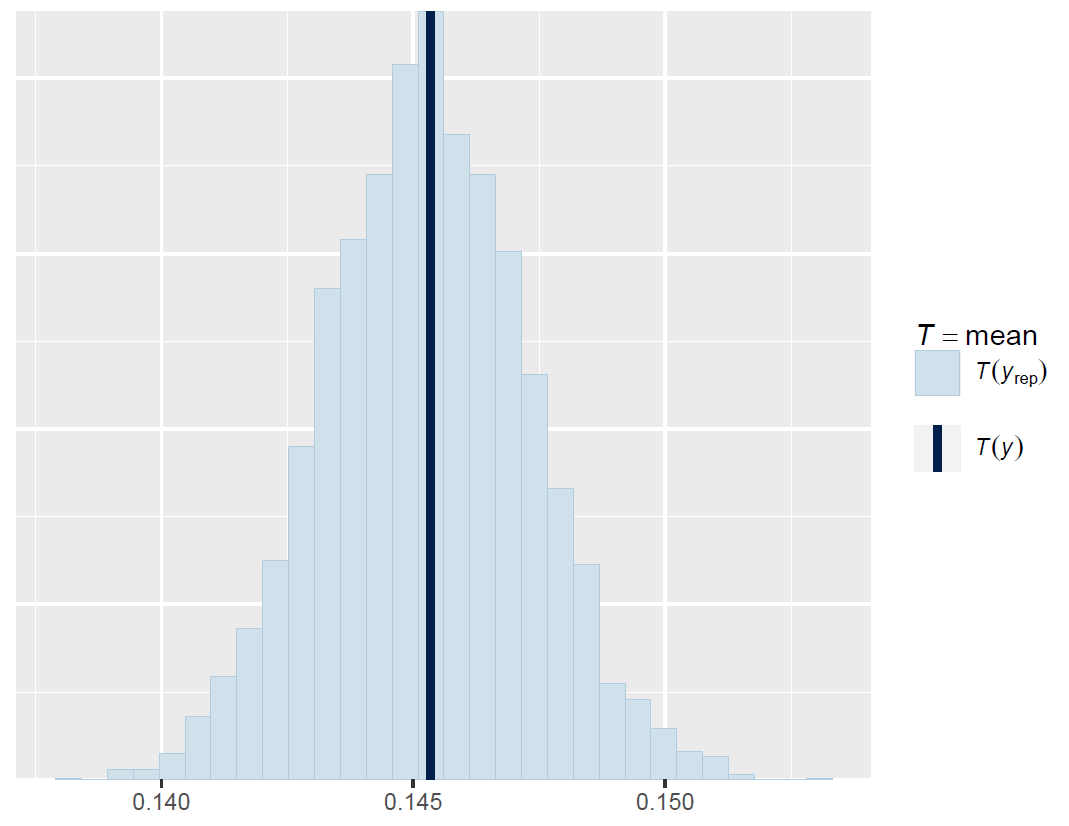


Figure S1. Histogram of the simulated distribution of the test statistic T(*y_rep_*), in this case the mean, computed from 4000 draws from the posterior predictive distribution with the observed value T(*y*) (mean).
